# Functional Abdominal Bloating with Distention

**DOI:** 10.5402/2012/721820

**Published:** 2012-06-19

**Authors:** Stephen Norman Sullivan

**Affiliations:** ^1^Department of Medicine, University of British Columbia, Vancouver, BC, Canada V5Z 1M9; ^2^Island Medical Program, University of Victoria, Victoria, BC, Canada V8W 2Y2

## Abstract

Ten to 25% of healthy persons experience bloating. It is particularly common in persons with the irritable bowel syndrome and constipation. While the cause of bloating remains unknown old explanations such as a excessive intestinal gas, exaggerated lumbar lordosis and psychiatric problems have been disproved. New suggestions include recent weight gain, weak or inappropriately relaxed abdominal muscles, an inappropriately contracted diaphragm and retained fluid in loops of distal small bowel. No treatment is of unequivocal benefit but a low FODMAPs diet, probiotics and the non-absorbable antibiotic rifaximin offer some hope. Treatment by weight loss, abdominal exercise, prokinetics and girdles need more study.

## 1. Introduction

Bloating is the symptom and distention is the sign. Not a pretty picture! What do we know about this highly prevalent problem and perhaps the most bothersome symptom for persons with the irritable bowel syndrome, especially of the constipated variety?

There have been several recent exhaustive reviews of the topic and important new research [1–15]. This is a brief summary of the relevant science and some of the art. For a historical perspective I highly recommend the classic paper by Alvarez of the Mayo Clinic [16].

Let me begin at the end and say that I believe that most cases of functional abdominal bloating with visible abdominal distention can be explained by some combination of weak or inappropriately relaxed abdominal muscles, a diaphragm that contracts when it should relax; excessive intraabdominal fat; fluid in loops of small intestine and gravity. I will review the evidence for my conclusions and for completeness include some other thoughts. 

## 2. Prevalence 

Visible, measurable, uncomfortable distention of the abdomen, “bloating,” may be due to aerophagy, gluttony, gastric outlet or intestinal obstruction, malabsorption, hypomotility of the stomach or intestine, obesity, or intraabdominal masses, and fluid. It may be a symptom of psychiatric conditions such as anorexia nervosa, bulimia, or the somatization disorder. If an organic or psychiatric cause cannot be found, then bloating is usually considered to be “functional” and part of the spectrum of the irritable bowel syndrome, functional dyspepsia, or the premenstrual syndrome. Unexplained abdominal bloating is a very common problem. In surveys of healthy individuals and populations 10–30% experience bloating often, frequently, or greater than a quarter of the time. 

## 3. The Clinical Picture

When a patient comes complaining of bloating make sure that you know what they are talking about. Only about half of patients who complain of bloating actually experience abdominal distention [8].

Functional abdominal bloating is never constant or unremitting. It comes and goes in one of two patterns—shortly after meals or near the end of the day. The former is often considered part of the spectrum of functional dyspepsia. For the purposes of this paper I will address only the latter.

The sufferer, usually a woman, awakens with a flat abdomen that progressively enlarges as the day goes on. Fasting or eating small meals minimizes the problem. Large or heavy meals make it worse as may constipation. It may be worse premenstrually. Loosening of the belt or changing of clothing may be necessary. Minor or temporary relief comes from burping, farting, or defecation. 

The distention is visible to the patient, family, friends, and the physician. It is measurable by tape, X-ray, computed tomography, and abdominal inductance plethysmography. With the miracle of digital photography some patients will come with photographic evidence or even post videos on *YouTube*. The skeptical physician may ask the patient to measure changes in abdominal girth, attend the office when the symptom is present, or email a photograph or video.

Many patients have symptoms of the irritable bowel syndrome, functional dyspepsia, constipation, or the premenstrual syndrome. They may also have psychological or social problems. Recent weight gain, weak abdominal muscles, and lack of exercise are common [17].

## 4. Etiology

To label a symptom “functional” begs the question, “Why does it occur?” There have been many suggestions (Table 1). I still remember the medical school mnemonic-food, fat, flab, fluid, flatus, feces, fetus, factitious, fatal, and fruitcake. The ten “Fs” were the causes of abdominal distention. The “fatal” referred to tumors and the “fruitcake” to patients with psychiatric problems. Which of the “Fs,” if any, might be an explanation for functional abdominal bloating?

A visible and measurable increase in abdominal girth implies an increase in intraabdominal volume. Intraabdominal contents increase with swallowed air, ingested food and fluid, retained feces and flatus, secretion of digestive juices, and possibly with increases in intraabdominal vascular volume during digestive and menstrual function. All of these events are normal but in some people cause “dis-ease” in the absence of disease.

Abdominal girth may also increase in the absence of an increase in volume if the abdominal contents are “redistributed” downwards and outwards. More on that later. First let us consider things that might increase volume.

## 5. Flatus

In veterinary medicine the term “bloat” refers to gaseous distention of the belly, sometimes necessitating treatment with a trocar. Such is not the case in human medicine. It has been repeatedly shown that human bloaters do not have “too much gas.” Farting and belching give only minor or transient relief and “anti-gas pills” are no better than placebo [18]. Bloaters may be excessively sensitive to normal amounts of gas or have trouble handling gas infused into their small intestine [10] or expelling gas infused into their colon [14] but when intestinal gas is measured by sophisticated CT scanning during bloating episodes there are only minor increases [4]. Visceral “hypersensitivity” has been invoked and may be a factor in patients who complain about bloating without abdominal distention as they are more sensitive to rectal distention than the bloaters with distention [7]. 

## 6. Food and Fluid

It is easy to understand why bloating might occur after meals. The increase in volume comes from food and fluids and the digestive juices produced in response. This sort of postprandial discomfort is labeled functional dyspepsia but my impression is that it is rarely associated with much abdominal distention. The topic of functional dyspepsia with its other symptoms is beyond the scope of this paper on visible bloating. 

This brings me to an idea that has not been adequately addressed, namely, fluid retention within loops of small bowel in the later postprandial period. If bloaters have trouble handing exogenous gas infused into the small intestine [10] could not they also have trouble handling endogenously produced secretions? After all, there are 6–8 liters being produced and absorbed each day. The “mishandling” of only a few liters could explain increased girth at the end of the day. Absorption overnight produces a flat abdomen in the morning. 

## 7. Feces 

Many constipated patients complain of bloating [8, 9] indeed difficulty in expelling a balloon from the rectum is predictive of bloating [15]. However the problem is not in the colon. The small volume of retained feces is not sufficient to increase abdominal girth. The problem seems to be “upstream.” Distention of the rectum slows proximal gut motility and some patients with constipation and distention have slow small intestinal transit [6, 19–22]. Even voluntary suppression of defecation slows gastric emptying [23]. Could slow proximal transit increase the volume of fluid within the small intestine?

## 8. Fat 

This is also an area that has not been fully explored. Most studies have not found a correlation between bloating and body mass index although having a BMI > 30 kg/m^2^ may be related [24, 25]. Bloaters do have a little bit of extra intraabdominal fat [4] but being obese should not cause excessive changes in postprandial or diurnal abdominal girth. However, a few kilos of extra intraabdominal fat might make one more sensitive to the normal fluctuations in intraabdominal volume that we all experience. Approximately 40% of patients with bloating have gained 10 or more pounds in weight in the preceding year [17] and about a quarter feel that their bloating problem began about the time they started to gain weight [9]. A substantial amount of recently gained weight is stored as intraabdominal fat [26] thus reducing space into which the abdominal contents can comfortably expand and perhaps causing the symptom of bloating.

## 9. Flabby Abdominal Muscles

Many years ago Osler noted that some patients with a bloating condition he called “enteroptosis” had “loss of the normal support of the abdominal wall” [27]. Some years later, Alvarez commented that some bloating patients had “relaxation of the muscles of the anterior abdominal wall” and suggested that some had a “neurosis" of the abdominal wall [16]. He may have been right. A third of patients whose primary symptom is visible abdominal bloating are unable to do even a single sit up [17]! And the most recent research has found that bloaters with distention have “impaired viscerosomatic reflexes and abdominal-wall dystony” with “incoordinated abdominal accommodation” and “abdomino-phrenic dyssynergia” leading to “caudo-ventral redistribution of contents” [4, 12, 13]. Simply stated, the abdominal muscles, especially the “anti-gravity” internal obliques, relax when they should contract and the diaphragm contracts when it should relax thus the abdominal contents sag downwards and outwards. This is nicely illustrated by the diagrams in [3, 4].

Redistribution of abdominal contents also occurs in women with severe vertebral osteoporosis and loss of abdominal vertical height. The rib cage rests on the pelvis and the abdomen protrudes because its contents have nowhere else to go.

## 10. Psychological Factors

The largest case series of functional abdominal bloating is still Alvarez's unfortunately titled “*hysterical type of nongaseous abdominal bloating*” [16]. His article began thus, “In 1911, I saw a psychopathic woman, past the menopause, who, with her protuberant abdomen, was sure she was pregnant by the Holy Spirit... I learned then that a bloated abdomen can be produced purely by nervous means.” Perhaps we can forgive his generalization, considering that it was the age of psychosomatic explanations for peptic ulcers and ulcerative colitis. 

Unfortunately it has too often been the habit of physicians to blame the patient's psyche for symptoms the physician cannot explain. Let us state categorically that the average functional bloater is not crazy or faking it! Some years ago my colleagues and I found that patients who had a consultation with a gastroenterologist for the primary complaint of unexplained visible abdominal bloating had the same degree of anxiety or depression and even a greater tolerance to pain as patients with quiescent Crohn's disease [28, 29]. And as for faking it, voluntary protrusion of the abdomen has a different CT appearance than spontaneous bloating and CT scans and lateral abdominal films have shown that women with IBS and abdominal bloating have a normal degree of lordosis and a normally positioned diaphragm [30]. Although recently the involuntary position of the diaphragm has been readdressed, it appears that as the abdominal contents sag downward and outward the diaphragm contracts rather than relaxes into a lower position, so-called abdomino-phrenic dyssynergia [4, 13]. The ancient observation that general anesthesia almost instantly relieves distention may now have an explanation—the diaphragm relaxes.

However, bloating is an extremely common symptom in apparently psychiatric conditions like anorexia nervosa, bulimia, and the somatization disorder. It is also true that persons who have experienced and survived extreme stresses such as the Nazi Holocaust frequently suffer from bloating [31]. Bloating, stress, anxiety, and depression may coexist with or aggravate functional disturbances such as the irritable bowel syndrome or functional dyspepsia. In two large population surveys bloating correlated with psychiatric dysfunction: depression, sleeping difficulties, problems of coping, panic disorder, and agoraphobia [32, 33]. However bloating is also a common symptom in healthy and normally noncomplaining persons, even runners and athletes [34, 35]. So even though many patients find that their bloating is worse when they are anxious or it correlates with depression, insomnia, difficulty in coping, or alcohol abuse, I do not think we should “reflexly” blame the psyche. A symptom may cause psychic distress. Psychic distress may take the patient to the physician but we should not assume that the psyche is the cause of the symptom.

## 11. Management 

The diagnosis of functional abdominal bloating can usually be safely made from history and physical examination alone. If a disease of sufficient likelihood to cause the problem cannot be excluded by history and physical then it should be excluded by selective investigation. Always a good idea is to rule out coeliac disease. 

Once an organic cause for the bloating has not been found and the patient has been reassured, science and tradition provide little proven course of action (Table 2). This topic has recently been reviewed [36]. There are no 101% proven treatments. The role of exclusion diets is uncertain. The patient has usually tried diet manipulation and is dissatisfied with the results. Certainly lactose, fructose, and sorbitol should be considered and some patients, even without coeliac disease, benefit from exclusion of wheat and rye [37] or going on a gluten-free diet. A FODMAP exclusion diet may be worth trying [38]. FODMAP stands for fermentable oligosaccharides, disaccharides, monosaccharides, and polyols. These are small molecules that are rapidly fermentable and osmotically active and could cause luminal distention. Probiotics and even nonabsorbable antibiotics like rifaximin are being studied [39, 40]. Weight loss may help but is unproven, as are girdles and abdominal exercises. Physical activity seems to help bloaters [41] and even runners and other athletes bloat less when exercising regularly [35, 36]. Perhaps walking or jogging will help the bloater. Laxatives help constipation and maybe the associated bloating. None of the “anti-gas” remedies are better than placebo although peppermint oil has some promise. If the problem is slow transit or extra fluid in loops of small intestine maybe a whole-gut prokinetic like cisapride, erythromycin or pyridostigmine will help [11]. Some believe psycho- or hypnotherapy are of benefit [42, 43]. 

Personally I recommend smaller meals, weight loss where appropriate, laxatives when needed, physical activity, abdominal exercises, and yoghurt. I used to try cisapride and tegaserod but they are no longer readily available. I might try erythromycin or pyridostigmine. I also recommend abdominal binders such as Slim Away seen on *YouTube*. As Osler noted, “I know of no single simple measure which affords relief to distressing symptoms in so many cases as the abdominal bandage” [27].

## Figures and Tables

**Table 1:** Suggested causes of functional abdominal bloating.

(1) Intra-abdominal gas	
(2) Fluid within the lumen of the gut	
(3) Feces	
(4) Low or inappropriately contracted diaphragm	
(5) Exaggerated lumbar lordosis	
(6) Intra-abdominal fat	
(7) Weak or inappropriately relaxed abdominal muscles	
(8) Psychiatric problems	

**Table 2:** Management of functional abdominal bloating.

(1) Exclude an organic cause
(2) Explanation and reassurance
(3) Diet manipulation
(4) Weight loss?
(5) Sit-ups?
(6) Exercise?
(7) A girdle?
(8) A laxative?
(9) A prokinetic?
(10) Probiotics?
(11) Non-absorbable antibiotics?
(12) Psycho- or hypnotherapy?
